# Research on Diagnostic Methods for Gas Generation Due to Degradation of Cable PVC Materials Under Electrical and Thermal Stress

**DOI:** 10.3390/polym17223021

**Published:** 2025-11-13

**Authors:** Peng Zhang, Xingwang Huang, Jingang Su, Zhen Liu, Xianhai Pang, Zihao Wang, Yidong Chen

**Affiliations:** 1 Hebei Electric Power Research Institute, State Grid Hebei Electric Power Co., Ltd., Shijiazhuang 050000, China; huangxingwang1997@163.com (X.H.); sujingang@tju.edu.cn (J.S.); lllzhen@163.com (Z.L.); dyy_pangxh@126.com (X.P.); 2 College of Electrical Engineering, Sichuan University, Wuhou District, Chengdu 610207, China; wangzihao1@stu.scu.edu.cn (Z.W.); 2022323030022@stu.scu.edu.cn (Y.C.)

**Keywords:** PVC, cable, molecule simulation

## Abstract

Polyvinyl chloride (PVC), owing to its excellent electrical properties and low cost, is widely applied in the inner insulation and outer sheath of cables. To achieve early fault warning based on characteristic gases, this study integrates experimental testing with molecular simulations to systematically reveal the decomposition and gas generation characteristics of different PVC layers under electrical and thermal stresses. The results indicate that inner-layer PVC under electrical stress predominantly generates small-molecule olefins and halogenated hydrocarbons, while outer-layer PVC during thermal decomposition mainly produces hydrogen chloride, alkanes, and fragments of plasticizers. The surrounding atmosphere significantly regulates the gas generation pathways: air promotes the formation of CO_2_ and H_2_O, whereas electrical discharges accelerate the release of unsaturated hydrocarbons such as acetylene. By employing TG-FTIR, ReaxFF molecular dynamics, and DFT spectral calculations, a normalized infrared spectral library covering typical products was established and combined with the non-negative least squares method to realize quantitative deconvolution of mixed gases. Ultimately, a diagnostic system was constructed based on the concentration ratios of characteristic gases, which can effectively distinguish the failure modes of inner and outer PVC layers as well as different stress types. This provides a feasible approach for early detection of cable faults and supports intelligent maintenance strategies.

## 1. Introduction

In recent years, cable fire incidents have occurred frequently due to factors such as line aging and overloading, causing severe losses to society and the economy [1]. Relevant statistics indicate that in China, more than half of major and catastrophic fires are triggered by cable faults, ranking first among all types of fire incidents, and resulting in significant casualties and property damage [2]. Polyvinyl chloride (PVC) cables, with their excellent electrical performance, good flexibility, and low cost, are widely used in construction, industry, and other fields. However, as service life extends, the insulation layer becomes prone to degradation under the combined influence of environmental and electrical stress, thereby increasing the risk of leakage, short-circuiting, and even fire hazards. Consequently, how to accurately monitor and provide early warning of potential cable defects before accidents occur, and how to take effective preventive measures, has become a critical issue urgently needing resolution in the fields of power systems and fire safety.

In the early diagnosis of cable faults, gas sensor technology has emerged as a research focus due to its high sensitivity and rapid response. Studies have shown that when insulating materials such as cross-linked polyethylene (XLPE) are subjected to overheating or partial discharge, they not only release carbon monoxide (CO) and carbon dioxide (CO_2_) but also generate characteristic gases such as methane (CH_4_) and ethylene (C_2_H_4_) [3]. These gaseous signals can serve as “fingerprints” of early-stage faults. Based on this principle, researchers have developed multi-channel or composite gas sensors capable of simultaneously detecting multiple gas components online. By integrating pattern recognition algorithms, these systems can achieve precise diagnosis and early warning of different fault types, including cable overheating, partial discharge, and insulation degradation.

However, compared with insulating materials such as XLPE, which have relatively simple structures, PVC cables exhibit a far more complex composition [4]. Current research has mainly focused on the thermal decomposition mechanisms of pure PVC materials [5]. In practice, however, there are numerous types of PVC with complex formulations, and significant differences exist among them in terms of the composition and dosage of additives. With respect to the decomposition characteristics of PVC materials in the cable industry—particularly their decomposition behavior under discharge conditions—relevant studies are still in an exploratory stage, and reports in the literature are scarce. Furthermore, in addition to the PVC matrix, cable-grade PVC materials are extensively compounded with a variety of additives such as plasticizers, stabilizers, and fillers. These additives exhibit markedly different degradation behaviors under varying temperatures and electric fields, resulting in extremely complex gas compositions when PVC cable faults occur [6,7]. In particular, the gas generation patterns of PVC materials under different atmospheric environments have not yet been thoroughly investigated, which severely limits a deeper understanding of the characteristic fault gases associated with PVC materials.

Although PVC cables are widely used in industrial and construction fields [8,9], research on their characteristic fault gases still remains at a preliminary stage, with reports on gas generation under discharge conditions being particularly scarce [10,11]. Current studies indicate that the thermal decomposition process of PVC cables can be broadly divided into three temperature ranges [12,13,14], with each stage producing distinct characteristic gases. In the low-temperature overheating stage (below 150 °C) [15,16,17], the decomposition of low-molecular-weight additives and moisture evaporation dominate, leading to the release of mainly water vapor and small amounts of volatile organic compounds. These gases are low in concentration and weak in characteristic specificity, making them unreliable indicators for fault diagnosis [18]. In the medium-temperature overheating stage (200–340 °C), the main chain of PVC begins to undergo significant degradation, releasing the typical characteristic product hydrogen chloride (HCl) [19,20]. Hydrogen chloride is highly corrosive and has a strong pungent odor [21,22], and it has become the hallmark gas for diagnosing medium-temperature overheating of PVC materials [23,24]. In the high-temperature overheating stage (above 400 °C), heat-resistant plasticizers begin to decompose, producing organic acids and aromatic hydrocarbons such as formic acid and toluene [5,25]. However, the gas composition at this stage is highly complex and present at high concentrations, and accurately distinguishing the sources of different gases and effectively diagnosing various fault types remains a pressing technical challenge yet to be resolved.

In addition, under high-energy discharge conditions, PVC cables may be subjected to the coupled effects of both thermal and electrical factors, resulting in decomposition mechanisms that differ significantly from those of purely thermal decomposition [26]. During the discharge process, local intensification of the electric field can cause the breaking and cross-linking of PVC molecular chains. At the same time, the rapid accumulation of heat further accelerates the thermal decomposition of the material. The gaseous products generated under such thermo-electrical coupling may differ markedly from those produced under conditions of purely thermal decomposition or electrical breakdown [27]. However, current research on the characteristic gases of PVC materials under discharge conditions remains extremely limited, which constrains the advancement of early diagnostic technologies for PVC cable faults.

In summary, current research on PVC cable fault diagnosis faces numerous challenges, among which the limited understanding of the stage-specific characteristics and mechanisms of gas generation from PVC decomposition under thermo-electrical coupling has become a critical bottleneck restricting the development of related diagnostic technologies [28,29]. In view of this, the present work adopts a combined approach of experimental investigation and simulation to conduct an in-depth study of the molecular-level decomposition mechanisms of PVC cables under the influence of thermal and electrical factors. Using techniques such as thermogravimetric analysis coupled with Fourier transform infrared spectroscopy (TG-FTIR), the study systematically analyzes the composition and variation patterns of evolved gases produced by PVC under different thermal and electrical stress conditions. Meanwhile, density functional theory (DFT) simulations are employed to elucidate the decomposition pathways and generation mechanisms of characteristic gases from a molecular perspective under thermo-electrical coupling. Through the mutual validation of experimental and simulation results, a database of characteristic gases associated with PVC faults is established, and its potential application in early fault diagnosis is explored. This research not only provides a solid scientific foundation for the early diagnosis of cable faults but also offers technical support for the development of novel gas sensors and intelligent diagnostic systems, thereby contributing to an effective reduction in the incidence of cable fire accidents.

## 2. Materials and Methods

### 2.1. Experimental Materials

**Figure 1:**
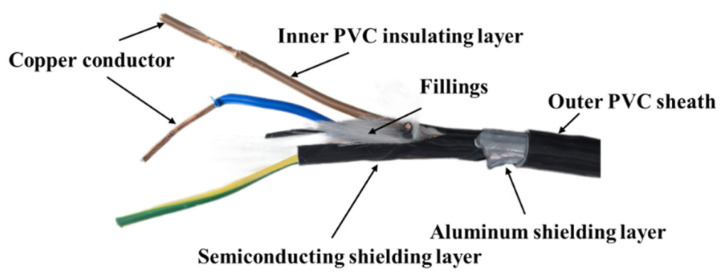
Structural schematic of a typical four-core cable.

The structural schematic of a typical four-core cable is shown in Figure 1. The PVC outer layer primarily serves as a barrier against moisture, while the PVC inner layer functions as insulation. The insulation materials used in the experiments were sample pieces stripped from the core insulation layer and the outer sheath layer of the cable. Cable stripping pliers were employed for sampling to ensure that the composition was consistent with that of the actual cable materials.

### 2.2. Experimental Equipment

Thermogravimetric–infrared (TG-IR) analysis was conducted using a thermogravimetric analyzer (TGA 2, Mettler Toledo, Greifensee, Switzerland) coupled with an infrared spectrometer (Foli20, INSA Optical, Shanghai, China). Fourier transform infrared (FTIR) measurements of gas products were performed using a gas IR spectrometer (Foli10, INSA Optical, China) equipped with a 5 cm optical path gas cell. Gas chromatography (GC) analysis was carried out on a gas chromatograph (GC112N, Shanghai Analytical Instrument Co., Shanghai, China) equipped with molecular sieve and alumina packed columns and fitted with flame ionization (FID) and thermal conductivity (TCD) detectors. The GC-MS (mass spectrometry) analyses were carried out using a Shimadzu GCMS-QP2020NX system equipped with an SH-I-5Sil MS capillary column (Kyoto, Japan).

### 2.3. Experimental Setup for Electrically Induced Gas Generation

**Figure 2:**
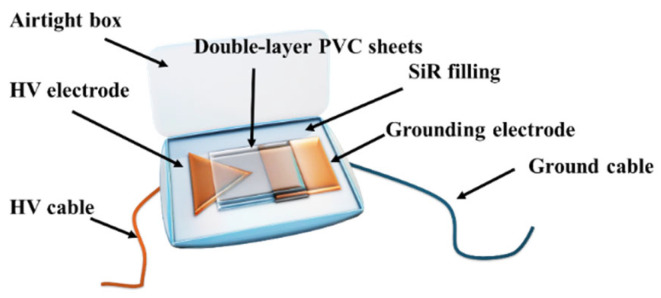
Diagram of the experimental device for electrically induced gas generation.

To clarify the gas generation characteristics of PVC materials under high-energy discharge conditions, an electrical breakdown experimental setup was designed, as illustrated in Figure 2. In the experiment, two PVC sample sheets were clamped between a pair of copper electrodes with a spacing of 5 mm and placed inside a silicone-sealed chamber to ensure good airtightness. To investigate the influence of different atmospheric environments on the gas generation process of PVC under electrical stress, high-purity air and argon were introduced separately into the chamber. During the experiment, a direct current (DC) voltage was applied to the electrodes, with the voltage increased stepwise at a rate of 1 kV/min until electrical breakdown occurred (at which point the power supply was immediately cut off). The gases generated during the breakdown process were collected using a syringe and subsequently analyzed by Fourier Transform Infrared (FTIR) spectroscopy.

### 2.4. Experimental Setup for Thermally Induced Gas Generation

To conduct an in-depth investigation of the thermal decomposition characteristics of PVC under different temperature conditions and atmospheric environments, a series of pyrolysis experiments were carried out using thermogravimetric-infrared coupling technology (TG-IR). The experiments employed a programmed heating mode in which PVC samples were gradually heated with a thermogravimetric analyzer (TGA), while an infrared spectrometer (IR) simultaneously tracked the real-time generation of volatile gases during the decomposition process. The experiments were performed separately under high-purity argon and air atmospheres, with a temperature range of 50–800 °C and a uniform heating rate of 50 °C/min. By continuously collecting infrared spectral data of the gases, the characteristic infrared absorption peaks at different temperature stages were analyzed in detail. Furthermore, to correct for the transmission delay error between TG and IR, calibration was achieved by aligning the IR peak temperature with the peak of the DTG curve. This ensured accurate elucidation of the gas release patterns and thermal degradation characteristics of PVC under pyrolysis conditions.

### 2.5. Molecular Simulation of Gas Generation from PVC

**Figure 3:**
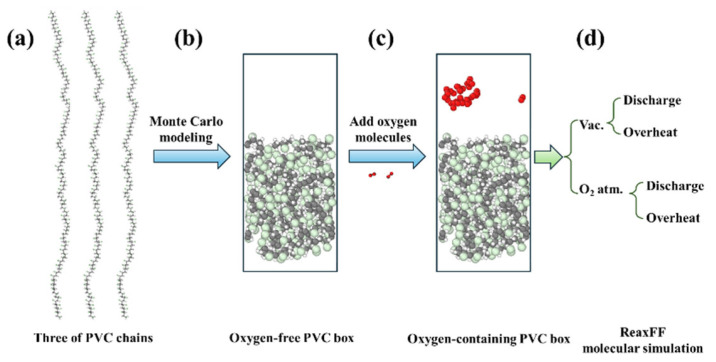
Schematic diagram of the PVC molecular simulation process. (**a**) Random construction of three PVC molecular chains; (**b**) folding of molecular chains using the Monte Carlo method; (**c**) introduction of oxygen molecules; and (**d**) ReaxFF molecular simulations under different influencing factors.

To further elucidate the decomposition mechanisms of PVC materials under the coupled influence of thermal and electric fields [18], a molecular simulation system was constructed as illustrated in Figure 3 [30,31]. First, a periodic model system containing three polyvinyl chloride (PVC, monomer unit: C_2_H_3_Cl) molecular chains was built using Packmol 21.1.1 software. The initial density was set at 1.38 g/cm^3^ to match the actual density of industrial PVC. Structural relaxation was then performed under the NPT ensemble at 300 K for 50,000 ps, ultimately ensuring stability in both density and conformation of the simulation system. Meanwhile, 40 oxygen molecules were introduced into the system to simulate real oxygen-containing environmental conditions. In addition, non-periodic boundary conditions were applied at the top and bottom surfaces of the system to realistically reflect the interfacial effects of PVC material surfaces.

For the simulation of the thermal field environment, precise temperature control was achieved using a Berendsen thermostat (built-in function in LAMMPS) with a damping coefficient of 25 fs. In view of the time-scale limitations of actual pyrolysis processes, the simulation temperature was appropriately elevated to 1250 K based on the principle of time-temperature equivalence, in order to accelerate the thermal decomposition process [32]. In simulating the effect of the electric field, the QTPIE method was introduced to model the charge polarization behavior of atoms within the system, while a direct current (DC) electric field of 30 kV/cm was applied along the Z-axis (converted to the atomic scale as 3.0 × 10^−4^ V/Å to ensure both stability and validity of the system). At the same time, considering the potential high-energy particle collision effects present under discharge conditions, a reactive force field suitable for describing interatomic reaction behaviors was adopted to simulate the molecular cleavage of PVC under realistic discharge conditions. The simulation temperature was likewise maintained at 1250 K.

To further identify the molecular vibration modes of characteristic gases generated during PVC pyrolysis, this study conducted quantum chemical vibrational spectroscopy calculations based on density functional theory (DFT). Specifically, the target gas molecular structures were constructed using Gaussian 16 software, and structural optimization as well as vibrational frequency calculations were performed using the B3LYP functional combined with the def2svp basis set. Since vibrational frequencies calculated by DFT are generally overestimated, a uniform frequency correction factor of 0.9671 was applied for wavenumber adjustments. Subsequently, the obtained spectra were broadened with Gaussian functions using Multiwfn (3.8(dev)) software. The calculated infrared spectra of the characteristic gases were then compared with experimental data to ensure the accuracy and reliability of the molecular simulations and computational analysis.

## 3. Results and Discussion

### 3.1. Analysis of Electrically Induced Gas Generation in PVC

**Figure 4:**
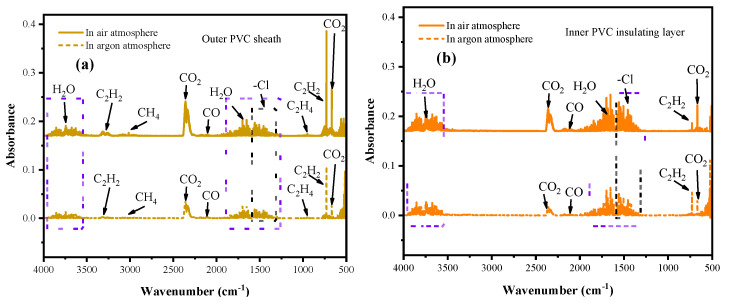
Infrared spectra of gases generated from the breakdown of the PVC outer layer (**a**) and inner layer (**b**) under air and argon atmospheres.

Gas infrared (IR) spectroscopy was employed to analyze the gaseous products released from the outer layer and the insulation layer of polyvinyl chloride (PVC) during electrical breakdown under air and argon environments. As shown in Figure 4a, the outer PVC layer produced substances including H_2_O, C_2_H_2_, CH_4_, C_2_H_4_, CO, and CO_2_, and chlorine-containing compounds. Among them, the peaks of chlorine-containing compounds overlapped partially with those of H_2_O (the chlorine-containing compounds being mainly hydrogen chloride), with small peaks of hydrogen chloride and methane appearing in the range of 2500–3000 cm^−1^. Compared with gas generation under argon, the peak intensities of all gases increased under air atmosphere, with particularly significant rises in acetylene and CO_2_. A comparison of Figure 4a,b reveals that the inner PVC insulation layer generated fewer types of substances during breakdown, mainly H_2_O, C_2_H_2_, CH_4_, CO, CO_2_, and chlorine-containing compounds. Compared with argon breakdown conditions, the CO_2_ peak increased under air atmosphere, whereas the acetylene peak decreased. Taken together, these results demonstrate that the types, concentrations, and proportions of gases generated by different PVC layers under different atmospheric conditions exhibit significant differences, and these gases can therefore serve as diagnostic criteria for distinguishing different fault types.

### 3.2. Analysis of Thermally Induced Gas Generation in PVC

**Figure 5:**
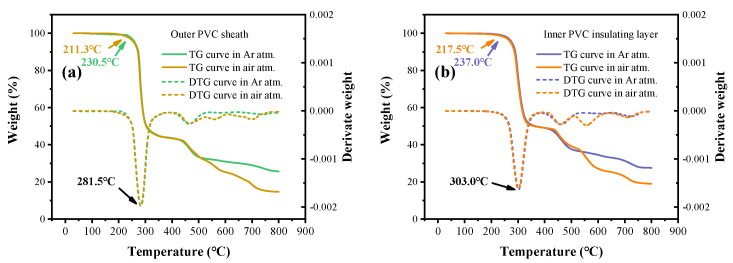
Thermogravimetric curves of the outer PVC layer (**a**) and inner PVC layer (**b**) under air and argon atmospheres.

The thermal resistance of the inner and outer layers of PVC shows significant differences. For the outer PVC layer (Figure 5a), the initial thermal decomposition temperature under argon is 230.5 °C, whereas under air it decreases to 211.3 °C, with the fastest weight-loss rate occurring at 281.5 °C. For the inner PVC layer (Figure 5b), the initial thermal decomposition temperature under argon is 237.0 °C, while under air it decreases to 217.5 °C, with the fastest weight-loss rate occurring at 303.0 °C. The thermal stability of both materials is notably reduced under an air atmosphere. This is because oxidation facilitates reactions that are difficult to occur under inert atmospheres, and the presence of oxygen decreases the stability of residual carbon, making it more prone to oxidation at elevated temperatures. Moreover, the gases released at these characteristic temperatures differ, corresponding to distinct temperature stages of thermally induced gas generation.

**Figure 6:**
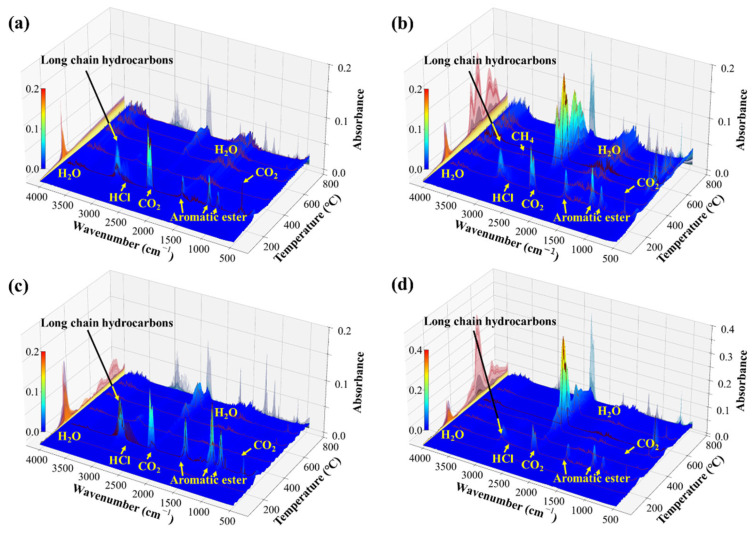
Three-dimensional temperature–infrared spectra of the outer PVC layer under argon (**a**) and air (**b**), and the inner PVC layer under argon (**c**) and air (**d**).

By analyzing the gas release characteristics with increasing temperature using thermogravimetric-infrared (TG-IR) techniques, the relationship between temperature and gas evolution under different atmospheric conditions can be established. Figure 6 illustrates the gas generation characteristics of both the outer and inner PVC layers in air and argon. It can be observed that the gases released differ significantly between the two layers and show clear temperature and atmosphere dependencies. Under inert atmospheres (Figure 6a,c), more HCl is produced, manifested as dense small peaks in the wavenumber range of 2500–3000 cm^−1^. In contrast, under air atmospheres (Figure 6b,d), a large amount of CO_2_ is generated in the temperature range of 400–800 °C, as certain carbon-containing substances are oxidized in air but remain stable under inert conditions. Overall, the outer layer releases a greater amount of aromatic ester gases (fragments of plasticizer molecules) but less hydrogen chloride compared to the inner layer. These aromatic esters originate from polyester-based plasticizers commonly used in PVC cables. Consequently, the characteristic gases can be used to determine whether overheating occurred in the inner or outer layer of the PVC cable, as well as to provide additional fault information such as air ingress, fault temperature, and severity.

### 3.3. Molecular Simulation of Gas Generation in PVC

To address the challenges that the radical reaction pathways during PVC decomposition are difficult to observe experimentally, and that the characteristic peaks of similar gases in mixed-gas infrared spectra are not easily distinguishable, this study employed reactive molecular dynamics simulations based on the ReaxFF method. This approach enables the characterization of gas generation behavior at the atomic scale under various influencing factors [33].

**Figure 7:**
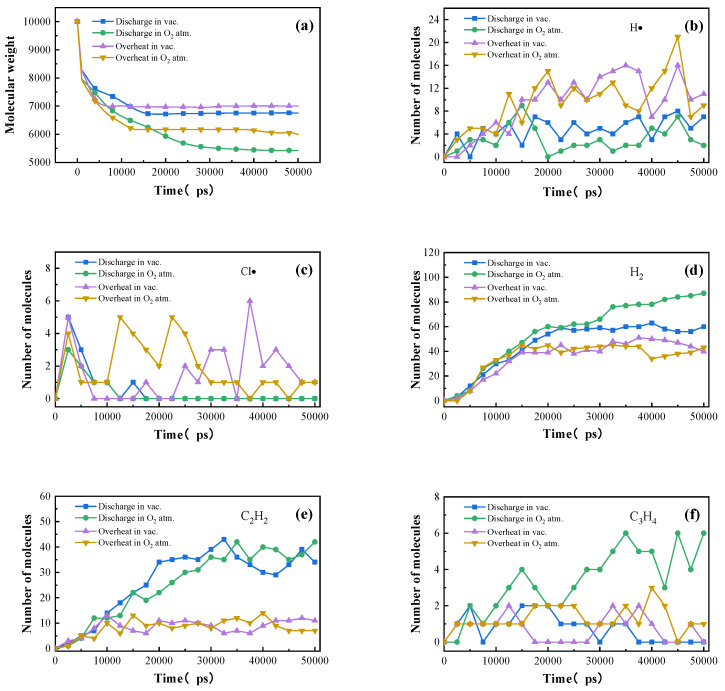
Variation curves of the molecular mass of solid residues of PVC under simulated discharge/overheating over time in vacuum and oxygen atmospheres (**a**), and the time-dependent evolution of hydrogen radicals (**b**), chlorine radicals (**c**), hydrogen (**d**), acetylene (**e**), and propyne (**f**).

Two types of statistical analyses were performed on the molecular dynamics simulation results. To simulate thermogravimetric weight loss, the total relative molecular mass of substances with molecular weights greater than 200 was calculated during the decomposition process of PVC. To simulate the evolution patterns of characteristic species, the number of gas molecules of individual substances was recorded as a function of time. As shown in Figure 7a, the decomposition behavior of PVC exhibited significant differences under simulated discharge and simulated overheating conditions. Under discharge conditions, the decomposition was extremely intense, with the residual solid mass rapidly decreasing. In contrast, under overheating conditions, owing to the presence of reversible reactions, the absence of electric-field acceleration of charged particles, and differences in the reaction potential energy surface, the molecular mass showed virtually no decline during the period from 15,000 to 50,000 ps. Furthermore, clear differences were observed in the gas generation rates under discharge and purely thermal conditions. The differences were particularly pronounced for acetylene, propyne, and hydrogen, with acetylene and hydrogen being generated at much higher rates under discharge than under purely thermal conditions. This finding is generally consistent with the experimental results, since decomposition under discharge is governed by the coupled effects of both electrical and thermal factors. The time-dependent generation rates of specific gaseous species are presented in Figure 7b–f. Under simulated overheating conditions, the period from 0 to 15,000 ps was dominated by end-group scission reactions, producing gases such as hydrogen, acetylene, chlorine radicals, and hydrogen radicals [34,35,36]. Among these, the yield of acetylene increased progressively over time (Figure 7e), while the concentrations of hydrogen, chlorine radicals, and hydrogen radicals increased rapidly in the 0–15,000 ps interval and then leveled off or declined. Specifically, hydrogen stabilized after its initial rise, whereas the concentrations of chlorine and hydrogen radicals began to decrease (Figure 7b–d). Under simulated discharge conditions, the generation of propyne exhibited a marked oxygen dependence: its yield remained steady in oxygen-free conditions but rose significantly in the presence of oxygen (Figure 7f). Overall, these simulation results are in good agreement with the experimentally observed decomposition characteristics of PVC [37,38,39].

### 3.4. DFT Calculation Results of Infrared Spectra of PVC Characteristic Gases and Establishment of a Spectral Database

To further analyze and quantitatively identify the characteristic gases generated during PVC decomposition, a normalized spectral database of characteristic gas infrared spectra was established through DFT calculations. Modern quantum chemistry enables relatively accurate calculation of the infrared spectra of individual molecules via DFT. The method employs the harmonic approximation (i.e., spring approximation) to simulate the primary vibrational modes of molecules and thereby obtain their vibrational frequencies and intensities (i.e., absorbance). The spectral database constructed using this approach contains spectra normalized to the same concentration, allowing subsequent algorithms to deconvolute mixed spectra and extract both the components of the mixture and the concentrations of individual constituents [40,41].

**Figure 8:**
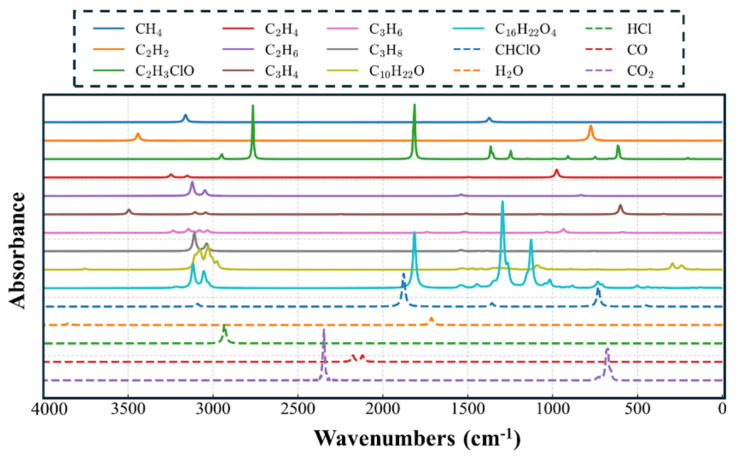
Normalized DFT-calculated infrared spectral library containing polyester plasticizer fragments, chlorine-containing compounds, and other typical small-molecule gases.

Figure 8 presents the infrared spectra results of several characteristic gases. Around 3000 cm^−1^ are the characteristic peaks of hydrocarbon gases, including the spectral peaks of long-chain alkanes with more than five carbon atoms, as well as ethane and propane. Near 2900 cm^−1^ appears the characteristic peak of HCl, along with chloroacetaldehyde, a typical compound containing chlorine, oxygen, and carbon. C_16_H_22_O_4_ corresponds to dibutyl terephthalate, representing the fragment peak of a major polyester plasticizer observed in thermogravimetric-infrared spectra. In addition, C_10_H_22_O corresponds to decanol, a typical long-chain product resulting from the decomposition of the PVC backbone. The normalized DFT spectral library established includes characteristic spectra of the main gases produced by PVC faults, thus providing a reliable foundation for quantitative concentration analysis of PVC-generated mixed gases and for fault diagnosis [42,43].

### 3.5. Quantitative Analysis and Fault Diagnosis of PVC

The Non-Negative Least Squares (NNLS) method is a commonly used and effective mathematical approach for quantitative peak deconvolution in the infrared spectroscopy of mixed gases. It minimizes the residual between measured and reference spectra while ensuring all fitted coefficients remain non-negative, thus preserving physical meaning. By fitting the experimentally obtained infrared absorption spectrum of mixed gases with the standard spectra of individual components, this method yields the optimal combination of spectral peaks. In this way, the quantitative characterization of different gas components can be achieved. Compared with the traditional linear least squares method, NNLS avoids physically unrealistic negative concentration results, thereby making the quantitative outcomes more reliable and physically meaningful.

**Figure 9:**
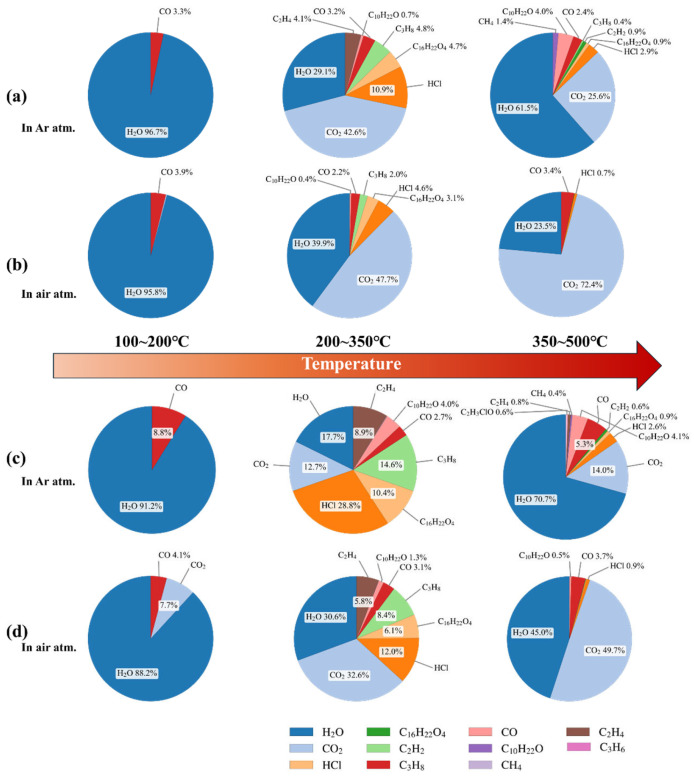
Proportion of gases in the outer PVC layer under argon (**a**) and air (**b**), and in the inner PVC layer under argon (**c**) and air (**d**) at different temperatures.

**Figure 10:**
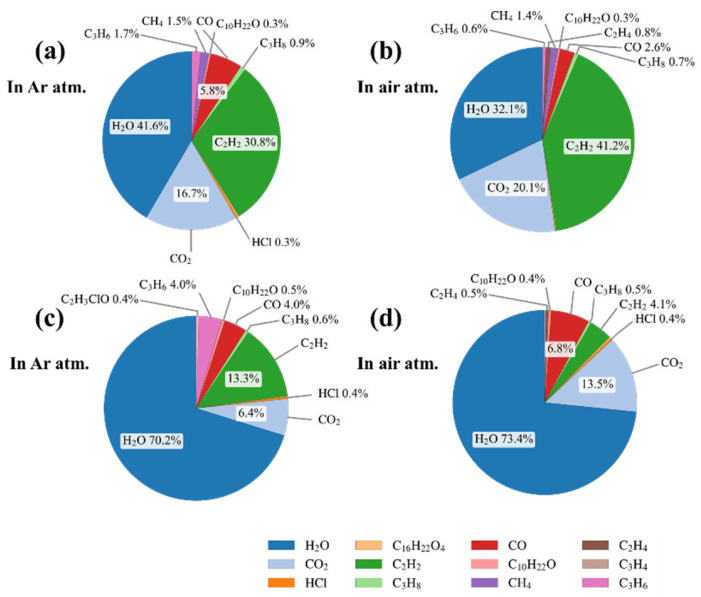
Proportion of gases generated under high-energy discharge from the outer PVC layer in argon (**a**) and air (**b**), and from the inner PVC layer in argon (**c**) and air (**d**).

By applying the Non-Negative Least Squares (NNLS) method in the analysis of mixed-gas infrared spectra, the concentrations of characteristic gases can be obtained, thereby allowing the calculation of their concentration ratios. The thermal gas generation characteristics of the two PVC materials under air and argon atmospheres at different temperatures are shown in Figure 9. In the low-temperature range (100–200 °C), the gases generated by heating both PVC materials were similar, consisting mainly of small amounts of water. Under argon, CO was the dominant gas, whereas in air both CO and CO_2_ were produced. When the temperature rose above the decomposition threshold (200–350 °C), a wide variety of gases were generated, including plasticizer fragments, alkanes, and hydrogen chloride, accompanied by significant CO release; under air, the proportions of CO and CO_2_ further increased. In the high-temperature range (350–500 °C), water vapor was the main product under argon, whereas under air CO_2_ dominated and the proportion of H_2_O decreased. At this stage, small amounts of highly unsaturated substances such as acetylene were also detected. The gas generation characteristics under high-energy discharge conditions are shown in Figure 10. Compared with thermal stress, discharge generated much larger amounts of acetylene. Moreover, under air atmosphere, more CO_2_ and H_2_O were produced compared with argon. The proportions of gases produced during discharge differed significantly between the two types of PVC, particularly for acetylene, ethylene, CO_2_, and H_2_O. By comparatively analyzing the concentration ratios of these characteristic gases, the decomposition characteristics of the two PVC materials under different fault conditions can be further clarified, thereby providing a quantitative basis for fault diagnosis and condition assessment.

**Figure 11:**
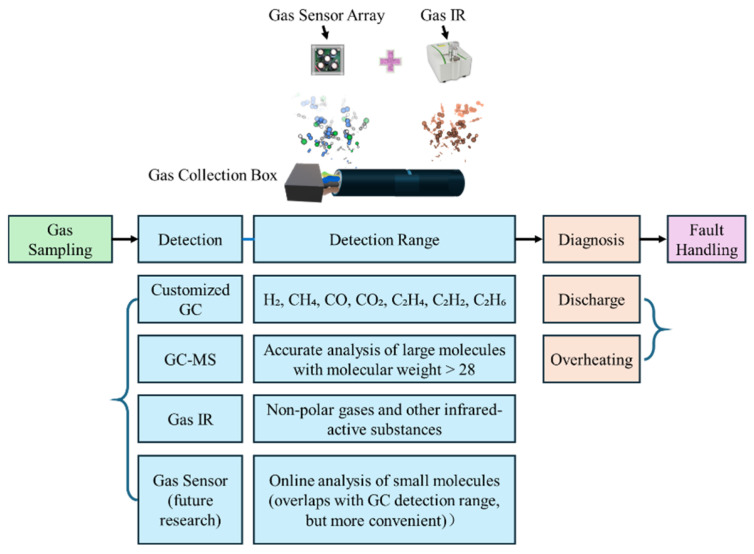
Workflow of gas detection and fault diagnosis for PVC cable degradation.

At the sampling stage, gases are collected at the cable termination using a sealed sampling box (or within the control cabinet) to capture fault-related emission gas. Figure 11 outlines the overall diagnostic process from gas sampling to fault handling. After gas sampling, the detection stage employs multiple analytical techniques: a customized GC system for common light gases (H_2_, CH_4_, CO, CO_2_, C_2_H_4_, C_2_H_2_, C_2_H_6_), GC–MS for accurate identification of large molecules (molecular weight > 28), and Gas IR for detecting non-polar and other infrared-active substances. Future work will involve gas sensors for online, real-time analysis of small molecules that overlap with the GC detection range but offer greater operational convenience. The measured gas composition and concentration information are then interpreted in the diagnosis stage to distinguish discharge and overheating faults, followed by appropriate fault handling strategies for early cable failure prevention.

Table 1 summarizes the characteristic gas products and their corresponding infrared absorption bands for the inner and outer PVC layers under different stress conditions and atmospheric environments. Under overheating, HCl is the major decomposition product in both layers, accompanied by alkanes, CO, and plasticizer fragments, with typical IR peaks around 2900 cm^−1^ (HCl, C–H stretch) and 1730 cm^−1^ (C=O of plasticizers). Under electrical discharge, unsaturated hydrocarbons such as C_2_H_2_ and C_2_H_4_ dominate, showing strong IR features near 730 cm^−1^ and 950–1000 cm^−1^. The atmospheric environment significantly modifies gas evolution: in air, oxidation leads to abundant CO_2_ (2350 cm^−1^) and H_2_O (3700, 1630 cm^−1^), whereas in argon, decomposition proceeds mainly through dehydrochlorination and chain scission, producing HCl and C_2_H_2_ as the key diagnostic gases. These spectroscopic signatures provide a direct basis for distinguishing between thermal and electrical degradation modes, as well as between oxidizing and inert environments in PVC cable fault diagnosis.

In future work, artificial intelligence (AI) is expected to play an important role in advancing this diagnostic system. First, AI algorithms such as neural networks and deep regression models can enhance the quantitative interpretation of spectral data, enabling more accurate conversion of infrared or sensor signals into gas concentration values, even under complex mixed-gas conditions. Second, by training on large datasets linking gas concentration patterns with specific fault types, AI can establish a more intelligent and adaptive fault recognition model, improving both diagnostic precision and real-time applicability in cable monitoring systems.

## 4. Conclusions

This study focused on the decomposition behavior of inner and outer PVC layers of cables under electrical and thermal stresses. By combining experimental observations with molecular simulations, the gas generation patterns of different structural layers and their environmental dependencies were systematically revealed. Through thermogravimetric–infrared (TG-FTIR) experiments, breakdown discharge experiments, and molecular dynamics together with DFT calculations, the decomposition pathways and generation mechanisms of characteristic gases in PVC materials under thermal, electrical, and thermo-electrical coupling conditions were comprehensively compared.

(1) Under thermal and electrical stresses, the two types of PVC materials exhibited distinct multi-stage decomposition mechanisms, with significantly different gas generation characteristics strongly influenced by the surrounding atmosphere. Air promoted oxidation reactions, resulting in greater CO_2_ and H_2_O production, while discharge generated acetylene and other unsaturated hydrocarbons.
(2) By integrating TG-FTIR experiments with ReaxFF and DFT molecular simulations, the radical reaction processes were elucidated, and the infrared characteristics of key products were verified. A normalized database of PVC decomposition gases was thereby established.
(3) By integrating experimental results, simulations, and spectral peak-fitting using the NNLS method, this study proposed a diagnostic system based on gas concentration ratios. This system enables accurate identification of key gases such as HCl, CO, CO_2_, and C_2_H_2_, effectively distinguishing between different failure modes of the inner and outer PVC layers, thereby providing a reliable foundation for infrared gas diagnosis.

In conclusion, this study not only provides a systematic understanding of the failure mechanisms of PVC cables under complex thermo-electrical environments but also establishes the theoretical and methodological basis for applying gas sensing and spectroscopic diagnostics in the early warning of cable faults. The findings of this research offer important reference value for the development of new intelligent monitoring devices and for enhancing the operational reliability of cables.

## Figures and Tables

**Table 1:** Diagnostic features and representative infrared absorption peaks of gases evolved from PVC under different stresses and atmospheres.

PVC Layer	Condition	Atmosphere	Diagnostic Features (Gas Types and Concentrations)	Infrared Peak Position (cm^−1^)
Outer layer	Overheating	–	HCl is the dominant gas, accompanied by alkanes, plasticizer fragments, and a small amount of CO at low temperature.	HCl ≈ 2900; CO ≈ 2100; CH stretch (alkanes) ≈ 2850–2960; C=O (plasticizer) ≈ 1730
Outer layer	Electrical discharge	–	C_2_H_2_ is the main gas, with CH_4_ and C_2_H_4_ also present; CO_2_ and H_2_O are nearly absent.	C_2_H_2_ ≈ 730 & 610; CH_4_ ≈ 3010; C_2_H_4_ ≈ 950–1000
Inner layer	Overheating	–	HCl is the primary product, with alkanes and a small amount of CO formed at low temperature.	HCl ≈ 2900; CO ≈ 2100; CH stretch (alkanes) ≈ 2850–2960
Inner layer	Electrical discharge	–	C_2_H_2_, CH_4_, and HCl are the main gases, while CO_2_ and H_2_O remain at very low levels.	C_2_H_2_ ≈ 730 & 610; CH_4_ ≈ 3010; HCl ≈ 2900
Overall criterion	Oxidizing atmosphere	Air	CO_2_ and H_2_O are the major products, accompanied by small amounts of CH_4_, C_2_H_2_, and CO. Oxidation promotes CO_2_ formation and reduces unsaturated hydrocarbons.	CO_2_ ≈ 2350; H_2_O ≈ 3700 & 1630; CH_4_ ≈ 3010; C_2_H_2_ ≈ 730; CO ≈ 2100
Overall criterion	Inert atmosphere	Argon	HCl and C_2_H_2_ are the main diagnostic gases. HCl indicates thermal decomposition, while C_2_H_2_ is typical of discharge-induced degradation under non-oxidizing conditions.	HCl ≈ 2900; C_2_H_2_ ≈ 730 & 610

## Data Availability

The original contributions presented in this study are included in the article. Further inquiries can be directed to the corresponding author(s).
